# Disability Certification Under the Rights of Persons With Disabilities (RPwD) Act, 2016 in Northeast India: An Institutional Analysis of Trends, Service Utilization, and Trauma-Related Locomotor Disability Burden

**DOI:** 10.7759/cureus.110545

**Published:** 2026-06-09

**Authors:** Bhaskar Borgohain, Sachlang Debbarma, Tashi G Khonglah, Chirag Sharma, Penthungo Ezung, Nebanshan Lyngdoh

**Affiliations:** 1 Orthopaedics, North Eastern Indira Gandhi Regional Institute of Health and Medical Sciences (NEIGRIHMS), Shillong, IND; 2 Orthopaedic Surgery, North Eastern Indira Gandhi Regional Institute of Health and Medical Sciences (NEIGRIHMS), Shillong, IND; 3 Orthopaedics and Traumatology, North Eastern Indira Gandhi Regional Institute of Health and Medical Sciences (NEIGRIHMS), Shillong, IND

**Keywords:** disability, locomotor disability, northeast india, rpwd act 2016, trauma

## Abstract

Background

Disability certification is a critical mechanism enabling persons with disabilities to access healthcare, rehabilitation, social welfare measures, and government entitlements under the Rights of Persons with Disabilities (RPwD) Act, 2016. Despite increasing global recognition of disability as a public health priority, data from Northeast India remain limited. This study aimed to evaluate temporal trends in disability certification applications, service utilization patterns, demographic characteristics, and trauma-related locomotor disability burden at a tertiary care center in Northeast India.

Methods

A retrospective review of disability certification applications submitted between January 2018 and November 2024 was conducted. Data regarding demographic characteristics, domicile, disability type, and severity were extracted from institutional certification records maintained by the Nodal Officer for disability certification. Applications were evaluated by the Institutional Medical Board according to RPwD guidelines.

Results

Of the 132 disability certification applications received between January 2018 and November 2024, 113 were included in the final analysis after excluding 19 incomplete records. Applications showed a progressive increase over time, peaking in 2023, with a temporary decline during the COVID-19 pandemic. Most applicants were male (77.0%) and belonged to the 19-35-year age group (38.9%). Applicants domiciled in Northeast India, predominantly from Meghalaya, constituted the majority of applicants. Physical disabilities accounted for most certifications, with locomotor disability being the predominant subtype. Trauma-related locomotor disabilities were observed particularly among working-age males. Moderate disability severity predominated, while visual impairment was predominantly observed among older adults.

Conclusion

The study underscores the increasing awareness and accessibility of disability certification services under the RPwD Act, particularly among rural and underserved populations. However, the low representation of individuals with mental illness, intellectual disability, specific learning disability, autism spectrum disorder, and multiple disabilities highlights the need for more inclusive and multidisciplinary assessment frameworks. Targeted outreach programs and community-based services are essential to address the potential underutilization of certification services in remote regions, particularly among individuals with trauma-induced disabilities.

## Introduction

Disability, as defined by the WHO International Classification of Functioning, Disability and Health (ICF), is a dynamic interaction between health conditions and contextual factors, resulting in impairments, activity limitations, or participation restrictions. Globally, disability affects a significant portion of the population. According to the WHO (2022), around 1.3 billion people, or 16% of the world’s population, live with a disability. An earlier WHO report (2011) estimated that 80% of people with disabilities reside in developing countries, where many continue to face widespread health inequalities [1,2,3]. In the Indian context, data from the National Family Health Survey-5 (NFHS-5), 2019-2021, reveal that 4.5% of individuals reported having a disability and 8.4% of households included at least one person with a disability. Among these, movement-related disabilities were most prevalent (44.4%), followed by mental health conditions (12.3%) and speech-related impairments (7.9%) [4].

The Rights of Persons with Disabilities (RPwD) Act, 2016, aligns with the UN Convention and recognizes 21 specified disabilities broadly grouped into physical disability, intellectual disability, mental behavior, disability caused due to chronic neurological conditions and blood disorders, multiple disabilities (more than one of the above specified disabilities), and any other category as may be notified by the Government [5]. Disability certification serves as an essential mechanism for official recognition, facilitating access to various support systems and benefits. To strengthen disability certification and service delivery, the Department of Empowerment of Persons with Disabilities launched the Unique Disability ID (UDID) project, which aims to establish a comprehensive end-to-end digital platform for issuing disability certificates and universal identification cards. The initiative promotes transparency, uniformity, efficiency, and streamlined access to government benefits for persons with disabilities [6].

Despite its importance, data from Northeast India remain limited, particularly in a region characterized by challenging geography, limited accessibility, and unique sociocultural diversity. This study aimed to evaluate temporal trends in disability certification applications, service utilization patterns, demographic characteristics, and trauma-related locomotor disability burden under the RPwD framework in a tertiary care setting in Northeast India.

## Materials and methods

Study design and setting

This retrospective observational study was conducted at a tertiary care teaching hospital and regional referral center in Northeast India, serving patients from Meghalaya and neighboring states.

Ethical approval

The study was approved by the Institutional Ethics Committee (Approval No. NEIGR/IEC/M2/F27/20240). A waiver of informed consent was granted due to the retrospective nature of the study and the use of anonymized secondary data.

Certification workflow and data collection

The institution has a multidisciplinary disability assessment and certification committee responsible for evaluating and certifying persons with disabilities under the RPwD Act, 2016. For the purpose of this study, records maintained by the Nodal Officer for disability certification were obtained following formal administrative approval. Extracted variables included demographic characteristics (age, gender, and place of residence), clinical characteristics such as type of disability, and certification parameters such as degree of disability.

Definitions and classification

The RPwD Act, 2016, recognizes 21 specified disabilities broadly grouped and subgrouped into physical disability (locomotor disability, visual impairment, hearing impairment, and speech and language disability), intellectual disability (specific learning disabilities and autism spectrum disorder), mental behavior, disability due to chronic neurological conditions and blood disorders, multiple disabilities (more than one of the above specified disabilities), and any other category as may be notified by the Government [5].

Severity was classified according to RPwD certification criteria as mild (<40%), moderate (40-80%), and severe (≥80%) disability, with ≥40% disability considered eligible for government benefits. Residence categories were classified as per the administrative records as rural, urban, or semi-urban.

Study population

All applications submitted for disability certification between January 2018 and November 2024 were included in the study. Both new applications and renewal cases were considered. Applications with incomplete or missing key data (age, gender, type of disability, or degree of disability) were excluded from the final analysis.

Statistical analysis

Data were analysed using Microsoft Excel. Categorical variables were summarized using frequencies and percentages.

## Results

Of the 132 applications received, all underwent administrative review; however, 19 applications containing incomplete demographic or disability-related information were excluded from the final statistical analysis. Statistical analysis was performed on 113 applications to assess temporal trends, demographic characteristics, and the distribution of disability categories.

Temporal trend (n = 132)

A progressive increase in applications was observed over time. Applications peaked in 2023, with 44 cases (33.3%), compared with lower numbers in the preceding years. A transient decline was observed in 2020, likely attributable to disruptions during the COVID-19 pandemic, followed by a rebound in the subsequent years (Table 1).

**Table 1:** Trend of applications received (n = 132). NA: Not applicable; December 2024 was not included because the study period ended in November 2024.

Month	2018	2019	2020	2021	2022	2023	2024
January	0	2	2	0	0	5	3
February	1	1	1	0	1	3	2
March	2	0	0	2	3	4	2
April	0	1	0	1	0	3	4
May	2	1	0	0	0	6	3
June	1	2	0	2	5	3	2
July	1	1	0	1	0	6	2
August	0	1	0	1	1	2	3
September	0	0	0	1	0	3	6
October	1	1	0	1	2	5	2
November	1	1	1	0	3	2	7
December	1	1	1	1	0	2	NA
Total	10	12	5	10	15	44	36

Demographic profile

Among the assessed applicants (n = 113), individuals aged 19-35 years formed the largest group, with 44 applicants (38.9%), followed by 33 applicants aged 36-55 years (29.2%), 28 applicants aged 0-18 years (24.8%), and 8 applicants aged above 55 years (7.1%). There was marked male predominance, with 87 males (77.0%) and 26 females (23.0%). Geographically, 85 applicants (75.2%) were domiciled in Northeast India; the majority were from Meghalaya, predominantly from rural areas, followed by Assam and Manipur. A substantial proportion of applicants in the non-Northeast domicile category were paramilitary personnel (Table 2).

**Table 2:** Demographic characteristics and disability categories of applicants (n = 113). Percentages were calculated using the total analysed sample (n = 113).

Variable	Category/subcategory	Frequency (n)	Percentage (%)
Age group (years)	0-18	28	24.8
	19-35	44	38.9
	36-55	33	29.2
	>55	8	7.1
Gender	Male	87	77
	Female	26	23
Domicile category	Northeast India	85	75.2
	Non-Northeast India	28	24.8
Disability category	Physical disability	83	73.5
	Intellectual disability	4	3.5
	Mental behaviour (mental illness)	0	0
	Disability due to chronic neurological conditions	13	11.5
	Multiple disabilities	1	0.9
	Any other category (unspecified)	12	10.6
Physical disability subcategory	Locomotor disability: trauma-induced	45	39.8
	Locomotor disability: non-traumatic	17	15.0
	Visual impairment	18	15.9
	Hearing impairment	3	2.7

Distribution of physical disability categories according to demographic characteristics and severity (n = 83)

Among individuals with physical disabilities (n = 83), a marked male predominance was observed, particularly among those with locomotor disabilities, where males accounted for 82.3% (51/62) of cases. This trend was even more pronounced in the traumatic locomotor disability subgroup, in which 91.1% (41/45) of affected individuals were male. Physical disabilities were predominantly observed in the economically productive age groups of 19-35 years and 36-55 years. Locomotor disability was most common in these age categories, accounting for 23 (37.1%) and 24 (38.7%) cases, respectively. Similarly, traumatic locomotor disabilities were concentrated in the working-age population, with 20 cases each occurring in the 19-35-year and 36-55-year age groups. In contrast, visual impairment was more frequently observed among older individuals, with 44.4% (8/18) of cases occurring in those aged over 55 years.

Regarding disability severity, moderate disability constituted the largest proportion of locomotor disability cases (44/62, 71.0%), followed by mild disability (19.4%) and severe disability (9.7%). Both traumatic (31/45, 68.9%) and non-traumatic (13/17, 76.5%) locomotor disabilities were predominantly classified as moderate in severity. Among individuals with visual impairment, mild disability was the most common category (11/18, 61.1%), while severe disability was present in 27.8% of cases. Hearing impairment cases were few and showed an even distribution across severity grades. Overall, physical disabilities in this cohort were characterized by a predominance of moderate-severity locomotor disabilities affecting working-age males, whereas visual impairment was more frequently encountered among older adults (Table 3).

**Table 3:** Distribution of physical disability categories according to demographic characteristics and severity (n = 83). Percentages for physical disability categories and subgroups were calculated using their respective denominators, including n = 83, n = 62, n = 45, n = 17, and n = 18, as applicable.

Category	Gender			Age group (year)				Disability severity		
	n	Male	Female	0-18	19-35	36-55	>55	Mild (<40%)	Moderate (40-80%)	Severe (≥80%)
1. Locomotor disability (musculoskeletal and movement-related disabilities)	62	51	11	13	23	24	2	12	44	6
Trauma-induced	45	41	4	4	20	20	1	10	31	4
Non-traumatic	17	10	7	9	3	4	1	2	13	2
2. Other disabilities in the physical disabilities category	21	15	6	4	4	5	8	12	3	6
Visual impairment	18	13	5	3	2	5	8	11	2	5
Hearing impairment	3	2	1	1	2	0	0	1	1	1

## Discussion

This study provides, to our knowledge, one of the first institutional analyses of disability certification patterns from Northeast India, offering region-specific insights into the implementation of the RPwD Act, 2016, in a geographically constrained and underserved setting. The findings extend the existing literature by demonstrating that disability certification trends are not merely reflective of disease burden but are strongly shaped by healthcare access, occupational exposure, and regional context.

Disability certificates are essential documents that serve as official proof of a person’s disability, enabling access to various government entitlements. As per the guidelines, individuals with 40% or more of a specified disability are eligible to avail of these benefits [5].

Our analysis reveals a consistent increase in applications for disability assessment services over time, with a transient decline observed during the COVID-19 period. The temporal increase in applications, with a transient decline followed by a rebound, further reflects the sensitivity of certification services to health system disruptions. The post-pandemic surge likely represents both the restoration of services and the addressing of previously unmet needs, emphasizing the importance of maintaining continuity of disability-related services during public health crises. This surge could indicate greater public awareness of available services and improvements in their accessibility. The trend aligns with findings by Nagarajan P et al., who also noted a rise in application volumes in their five-year trend in issuing disability certificates from a general hospital psychiatric unit in South India [7].

The demographic profile of the applicants reveals a male predominance, mirroring findings from previous studies conducted in North India [8]. The under-representation of female applicants in the present study may reflect broader gender disparities in healthcare access and utilization. Sociocultural factors, limited awareness, financial dependency, mobility constraints, and reliance on caregivers may reduce access to disability certification services among women, particularly in rural settings. In addition, a significant number of applications received were from rural and semi-urban areas. This high proportion of rural applications reflects both improved outreach under the RPwD framework and the persistent dependence of underserved populations on tertiary care centers for certification services. This also reflects the RPwD Act, 2016’s push for inclusive access in underserved regions, aligning with Saikia N et al.’s findings that nearly 70% of India’s disabled population resides in rural areas [9]. However, this pattern raises important concerns regarding geographical inequities, as individuals in remote areas may access certification only after significant delays or advanced disability. These findings reinforce the need for decentralized and community-linked certification models, including mobile assessment units and strengthened primary-level screening.

Looking at the paramilitary applicant perspective, the literature suggests that military service and disability are closely interconnected, as personnel engaged in active duty are frequently exposed to physically demanding environments, high-risk operational settings, repetitive mechanical stress, and traumatic events, all of which may contribute to long-term physical and psychological morbidity [10]. Previous studies have demonstrated that military and combat-related occupations are associated with an increased risk of disability and early retirement. Niebuhr DW et al. reported that combat exposure and physically intensive roles were linked to higher disability retirement rates, particularly due to musculoskeletal disorders, injuries, and mental health conditions [11]. In the present study, paramilitary personnel constituted a notable proportion of applicants from outside Northeast India, highlighting an important yet underexplored dimension of disability certification, namely occupational and service-related disability. The findings suggest that disability certification systems may serve not only as welfare mechanisms but also as important platforms for occupational health monitoring, rehabilitation, and reintegration of high-risk professional groups.

The detailed breakdown of disability types in the present study highlights the diversity within the assessed population. The RPwD Act, 2016, recognizes 21 specified disabilities, broadly grouped and subgrouped into physical disability (locomotor disability, visual impairment, hearing impairment, and speech and language disability), intellectual disability (specific learning disabilities and autism spectrum disorder), mental behavior, disability due to chronic neurological conditions and blood disorders, multiple disabilities (more than one of the above specified disabilities), and any other category as may be notified by the Government. In our cohort, physical disabilities predominated among all disabilities, accounting for 73.5% (83/113), of which locomotor disabilities accounted for 54.9% (62/113), a rate exceeding that reported in previous studies conducted in rural Jodhpur by Mishra K et al. [12]. Additionally, the majority of applicants with locomotor disability were male and in the 36-55-year age group, aligning with findings from the National Sample Survey Office and prior studies by Mahmood SE et al. and Suganthi S and Kandaswamy M [13,14].

A key observation is the predominance of locomotor disabilities in our cohort, which comprised both trauma-related etiologies, such as amputations, fractures, and post-traumatic deformities, and non-traumatic etiologies, such as neurological conditions, including cerebral palsy, and congenital conditions. The prominence of trauma-associated conditions suggests a considerable trauma-related contribution to the disability burden within the studied population, in contrast to the findings of Bora MH and Chetia NP, which indicated that 95% of disabilities were of musculoskeletal origin and acquired conditions [15]. This trauma-dominant pattern differs in magnitude from reports in other parts of India and suggests a substantial, potentially preventable burden of disability in the region. The findings highlight the intersection between injury epidemiology and disability certification, underscoring the need for integrated trauma care systems, early rehabilitation, and preventive strategies, particularly in geographically remote areas where delays in care may exacerbate long-term disability.

A novel and important finding of this study is the near absence of intellectual, neurodevelopmental, and other non-visible disabilities in certification records, despite the well-established prevalence of these conditions in the general population. In our study, this observation may likely reflect under-identification, delayed referral, limited specialist availability, and underutilization of certification services rather than a true epidemiological absence. Additional contributory barriers may include inadequate awareness, sociocultural stigma, and restricted access to multidisciplinary evaluation. These findings highlight a critical limitation of the existing certification system and underscore the need for comprehensive multidisciplinary assessment frameworks integrating psychiatry, pediatrics, psychology, and rehabilitation services to ensure equitable recognition and certification of non-visible disabilities.

Although derived from a single tertiary-care institution, the findings may have relevance to other geographically remote and resource-limited settings facing similar challenges in disability service delivery.

From an institutional perspective, tertiary care institutions serve not only as centers for standardized disability certification but also as important sources of regional epidemiological data. Systematic analysis of institutional certification records can facilitate the development of region-specific disability registries, providing valuable insights into local disability patterns and service needs. Such data are essential for evidence-based healthcare planning, equitable resource allocation, strengthening rehabilitation services, and monitoring policy implementation, particularly in underserved and geographically challenging regions such as Northeast India.

Limitations

The present study has certain limitations that should be acknowledged. The retrospective design, relatively small sample size, and single-center setting may limit the generalizability of the findings. As the study included only individuals attending a tertiary care disability certification service, the observed distribution does not represent true community prevalence and may be influenced by referral patterns and healthcare accessibility. Variables such as awareness regarding certification benefits, accessibility barriers, sociocultural stigma, delayed referrals, and unmet rehabilitation needs were not directly evaluated; consequently, patterns in service utilization were inferred from certification trends. Furthermore, the inclusion of an unspecified disability category may reduce interpretability because of incomplete subclassification within institutional records. Although temporal trends were examined, the findings remain primarily descriptive and should be interpreted within the context of an institutional tertiary care dataset.

## Conclusions

This retrospective institutional study highlights increasing utilization of and awareness of disability certification services under the RPwD Act in Northeast India, including among rural and underserved populations. Physical disabilities, particularly locomotor disabilities, were the predominant categories, with trauma-related conditions contributing substantially. In contrast, the low representation of intellectual disabilities, neurodevelopmental disabilities, mental illness, autism spectrum disorder, and multiple disabilities suggests possible under-identification, delayed referral, limited specialist access, and underutilization of certification services. As the findings are derived from a tertiary care referral population, they may not reflect community prevalence or unmet disability needs. The study emphasizes the need for inclusive, multidisciplinary disability assessment frameworks integrating psychiatry, pediatrics, neurology, and rehabilitation services, along with strengthened outreach, early referral pathways, and community-based services in geographically challenging regions.
